# DFT study of structural, elastic, electronic and dielectric properties of blue phosphorus nanotubes

**DOI:** 10.1038/s41598-019-47764-7

**Published:** 2019-08-02

**Authors:** Junhua Hao, Zhengjia Wang, Qinghua Jin

**Affiliations:** 1 Department of Physics, Tianjin University Renai College, Tianjin, 301636 People’s Republic of China; 2 Condensed Matter Science and Technology Institute, School of Instrumentation Science and Engineering, Harbin Institute of Technology, Harbin, 150080 People’s Republic of China; 3 School of Physics, Nankai University, Tianjin, 300071 People’s Republic of China

**Keywords:** Physical chemistry, Condensed-matter physics

## Abstract

Because of the flexibility band structure, the nanotubes based on the (001) two-dimensional monolayer of *β*-P are expected to be a promising candidate for electronic and optical applications. By density functional theory calculations, it could be investigated the structural stability of single-wall armchair and zigzag blue phosphorus nanotubes. The formation energy, structure parameter, Young’s modulus, radial Poisson’s ratio, band gap and static electronic polarizabilities for the two types of nanotubes are computed and analyzed as functions of the tube radius and axial strain. The properties of armchair and zigzag nanotubes are almost the same, and isotropy is observed for radius up to 13 Å. Furthermore, the band gaps are sensitive to the effects of axial strain.

## Introduction

Graphene is a flat monolayer of carbon atoms tightly packed into a honeycomb sheet, which has a zero band gap and a unique massless Dirac-like electronic excitation^1,2^. Since the discovery of graphene in 2004, two-dimensional (2D) nanostructures (e.g. graphene, silicene, MoS_2_, hexagonal BN) have attracted a great deal of attention in nanoelectronic devices due to their distinct structural and electronic properties^3–14^.

Recently, phosphorene, another stable two-dimensional elemental matter, which also possesses a hexagon skeleton like graphene, was exfoliated by mechanically cleaving the bulk black phosphorus (BP). Unlike graphene, phosphorene exhibits a puckered non-planar structure and has an inherent band gap^15–25^. Also, the band gap increases with a decreasing number of layers and is ~0.9–1.0 eV for monolayer. These properties are superior to graphene and some other 2D materials, which will open a door for application of optoelectronics.

The P atoms in phosphorus possess a hybridization sp^3^ state, so there are many allotropes of phosphorus such as α-P (black), β-P (blue), γ-P, and δ-P^26–31^. Among these structures, the monolayer of blue phosphorus has a small out-plane hexagonal structure which is very close to the structure of graphene^32–34^. Furthermore, it has a wider indirect energy gap of more than 2 eV. Due to its structure and properties, blue phosphorus will become a worthy contender used in electronic devices.

Tunable electronic properties of 2D materials will make them more attractive. Converting a two-dimensional slab to one-dimensional (1D) nanotube is a common strategy. In the past decade, the discovery and synthesis of carbon nanotubes, silicon nanotubes, MoS_2_ nanotubes and BN nanotubes have opened a promising and dynamic new field in condensed matter physics and chemistry due to their remarkable properties and a wide range of potential application. Very recently, Aierken and Montes *et al*. have investigated the properties of faceted blue phosphorene nanotubes (PNT) and the stability of blue PNT with different temperature by first-principle calculations^35^. By applying appropriate uniaxial or biaxial strains, Fei and Yang have demonstrated a new mechanism to engineer unique anisotropic conductance in single and few layers of black phosphorus^16^. However, the properties of blue PNT such as charge transfer, band gap, Young’s modulus, Poisson’s ratios under strain still need to be further studied.

**Figure 1:**
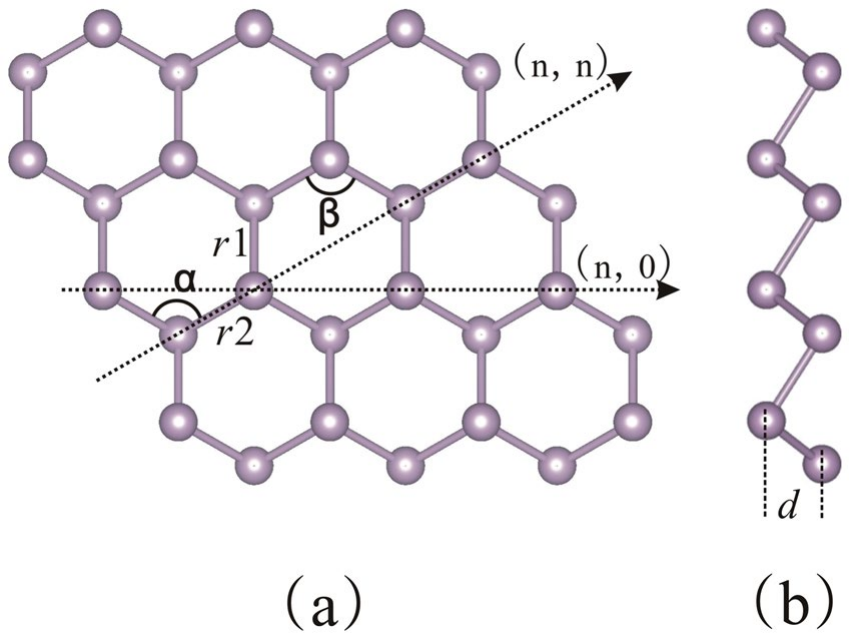
The optimized structure of blue phosphorus monolayer in (**a**) top and (**b**) side views.

The phosphorus allotrope of blue P shows the same stability as black P under normal conditions^26,36^. The crystal was described in the space group $P\bar{{\rm{3}}}\mathrm{m1}$ with two atoms per unit cell. By using the keyword SLABCUT, it can be obtained a 2D blue P sheet “cut” from the 3D crystal, as illustrated in Fig. 1. To compare with the black P monolayer and graphene, we first computed the structural parameters of blue P monolayer, as shown in Table 1. It can be seen that the atoms of blue P monolayer appear smoother than black P monolayer which is closely related to graphene. Compared with the other two sheets, the wide fundamental band gap is the main advantage of blue P monolayer. Based on GGA calculations, it is known to underestimate the band gap, which often results in lower measurements than experimental values. The 1D nanotubes are constructed based on the two-dimensional hexagonal structure (001) slab rolling, and then a cylindrical coordinate system is introduced.

**Table 1:** Structural parameters of blue P monolayer, black P monolayer and graphene.

Parameter	Blue P Monolayer		Black P Monolayer		Graphene
	Our work	ref.^32^	ref.^24^	ref.^25^	refs^3,4^
*r*1 [Å]	2.30	2.27	2.26	2.22	1.42
*r*2 [Å]	2.30	2.27	2.31	2.26	1.42
*d* [Å]	1.27	1.24	2.17	2.51	0
α [°]	92.58	92.88	95.90	95.9	120
*E*_g_ [eV]	2.07	1.94	0.9	~1	Semimetal
σ_ac_ (σ_zz_)	0.114		0.22 (0.76)		0.16
*Y*_ac_ (*Y*_zz_) [GPa]	136		43.5 (176.5)		1100
*C* [J·m^−2^]	439		445		335

The bond lengths (*r*1, *r*2), puckered-layer distance (*d*), and bond angle (α) of the relaxed blue P monolayer are described in Fig. 1.σ_ac_ (*Y*_ac_) and σ_zz_ (*Y*_zz_) are the Poisson’ ratio (Young’s modulus) of slab along zigzag and armchair directions, respectively. Energy gap (*E*_g_) and in-plane stiffness (*C*) are given.

In this work, the properties of single-wall armchair and zigzag blue PNT are investigated based on first-principles calculations within density functional theory (DFT). The lattice parameter, band gap, Poisson’s ratios, Young’s modulus, and the in-plane stiffness of blue P sheet are obtained and then compared to the other sheets (black P sheet and graphene) in detail. Here, it is also shown that the properties of blue PNT with the effects of radius and external strain systematically.

## Results and Discussion

**Figure 2:**
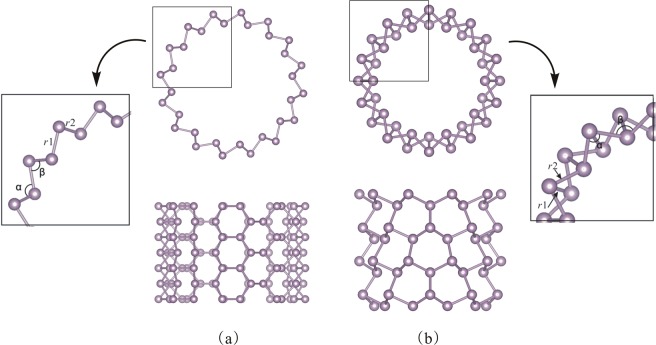
Transversal (top) and longitudinal (bottom) views of the (10, 10) armchair and (14, 0) zigzag blue PNTs in (**a**,**b**), respectively.

By rolling up the monolayer along the vector ${\boldsymbol{r}}={\rm{n}}{\boldsymbol{a}}+{\rm{n}}{\boldsymbol{b}}$ (5 ≤ n ≤ 24), it generates the armchair (n,n) blue PNTs with *R* ranging from 4.67 Å to 22.03 Å and zigzag (n,0) blue PNTs with *R* ranging from 2.96 Å to 12.76 Å. Due to the buckling, the mean values of radii of the two different coaxial cylindrical surfaces are selected for the nanotubes’ radii *R*. Top views, and side views for the armchair (10,10) and zigzag (14,0) nanotubes are displayed in Fig. 2a,b, respectively.

**Figure 3:**
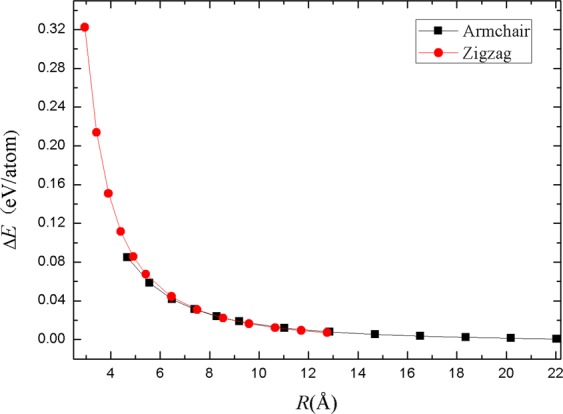
The formation energy with respect to the radius of blue PNTs.

The formation energy *E*_*f*_ of the optimized tubes is defined as ${E}_{f}=E({\rm{nano}})-E({\rm{slab}})$, in which *E*(nano) and *E*(slab) denoted the monoatomic energy of the nanotube and phosphorene slab. Our results are pictured in Fig. 3 which by the classical theory of elasticity and follow a *R*^−2^ law^37^. For the radius smaller than 8 Å, the formation energy increases dramatically as the nanotube radius decreases.

**Figure 4:**
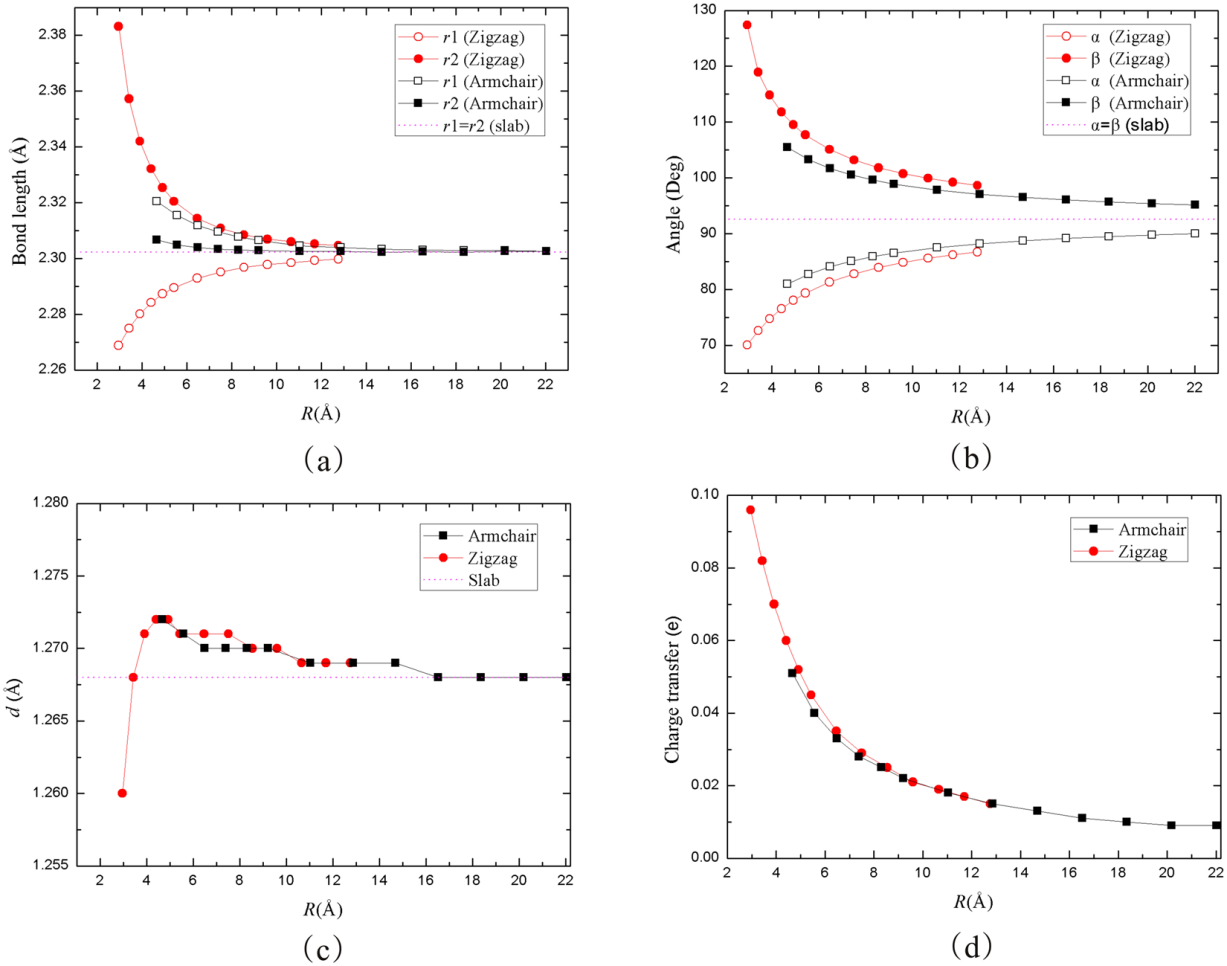
(**a**) Bond lengths of the armchair and zigzag blue PNTs are plotted versus tube radius. (**b**) Angles of two types of blue PNTs as a function of radius. (**c**) Dependence of puckered-layer distance *d* of blue PNTs with different radius. (**d**) The amount of charge transfer between P atoms in blue PNTs with different radius. The dashed horizontal line denotes the values of the corresponding blue phosphorene slab.

After the structure fully relaxed, as depicted in Fig. 4., the bond length, angle, puckered-layer distance, and charge transfer with different radius at zero strain were obtained. The bond length *r*1 equals *r*2 in the blue P sheet. Due to the non-planar honeycomb-like structure, the corresponding equivalence of the bond length *r*1 and *r*2 are broken when the sheet rolls into a nanotube. As shown in Fig. 4a, the degree of the bond lengths *r*1 and *r*2 are both elongated with the radius decreasing for armchair blue PNTs. *r*2 is changing more evidently than *r*1. However, the bond length *r*1 is contracted and *r*2 is elongated in zigzag blue PNTs. All of the bond lengths depend on the nanotube radius and finally converge to that of blue P sheet. The change of the bond lengths can be explained by the curvature that leads to a P-P orbitals rehybridization in nanotubes.

Meanwhile, the angles between the bonds are also changing with the tube radius. There are two angles (α and β) examined in our work, as shown in Figs 2 and 4b. The β value of two blue PNTs decreases with the increase of radius, while the α value increases with the increase of radius. With the same radius, the absolute value of cure slope for the zigzag blue PNTs are larger than the armchair blue PNTs. And they all converge to the value of the sheet’s angle when the radius is large enough. The puckered-distance *d* is presented in Fig. 4c. The separation is hardly noticeable for both types of blue PNTs and finally converges to the monolayer ones. Additionally, it could be obtained that the charge transfer for the tubes by Mulliken population analysis (see Fig. 4d)^38^. The charge transfer from the outer shell to the inner shell increases as the radius decreases. This is owed to the covalent interactions as the P-atoms approach.

**Figure 5:**
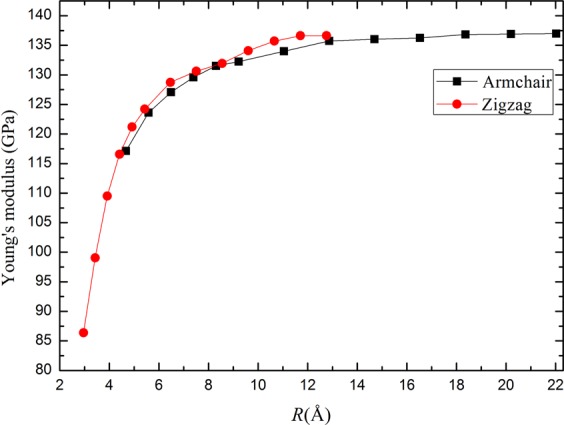
The dependence of Young’s modulus for PNTs on radius.

Besides the structure parameter, the mechanical properties of blue PNTs are also investigated. Young’s modulus, *Y*, has been calculated by the second order derivative of the total energy *E*_Tot_ with regard to the axial strain *ε* at *ε* = 0, given by

$$Y=\frac{1}{{V}_{0}}{(\frac{{\partial }^{2}{E}_{{\rm{Tot}}}}{\partial {\varepsilon }^{2}})}_{\varepsilon =0}$$

where *V*_0_ is the equilibrium volume and defined as ${V}_{0}=2{\rm{\pi }}R{L}_{0}\delta$; *R* is the tube radius; *L*_0_ is the tube length at *ε* = 0; *δ* = 5.6 Å is the shell thickness which is often chosen as the van der Waals distance^26^; $\varepsilon =(L-{L}_{0})/{L}_{0}$ is the axial strain. We performed calculations for the same tube under axial stress and optimized the coordinates of the atoms until minimum energy is obtained. Using the strain-energy relation, Young’s modulus of the tubes can be deduced from the second derivative of the stress at zero strain. The results are shown in Fig. 5. Obviously, Young’s modulus of nanotubes with small radius (*R* < 13 Å) increases with the increase of tube diameter. For a given radius, Young’s modulus of zigzag blue PNT is slightly larger than the armchair one. Then both of them will approach the value obtained for the blue P sheets when the tube radius larger than 13 Å. Similar to the blue PNTs, the effect of tube stiffing with increasing radius is also found in black PNTs, AlN nanotubes and ZnO nanotubes with small radius^24,39,40^.

**Figure 6:**
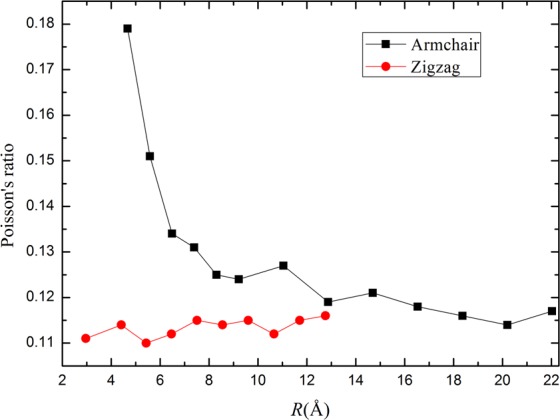
Poisson’s ratio versus radius.

Another interesting mechanical property is Poisson’s ratio *σ*, which is defined as

$$\sigma =-\,\frac{1}{\varepsilon }(\frac{R(\varepsilon )-R(0)}{R(0)})$$

where *R*(*ε*) is the radius of the tube at the strain *ε*, and *R*(0) is the radius of the unstrained tube (*ε* = 0). The Poisson ratios for various tubes are depicted in Fig. 6. It illustrates that the Poisson ratio of armchair blue PNTs is slightly larger than zigzag ones when the radius is small. As the radius increases, the values of the Poisson ratio for two types of blue PNTs will approach the same value. The results indicate that 2D blue phosphorene structure has isotropic properties along zigzag and armchair directions.

**Figure 7:**
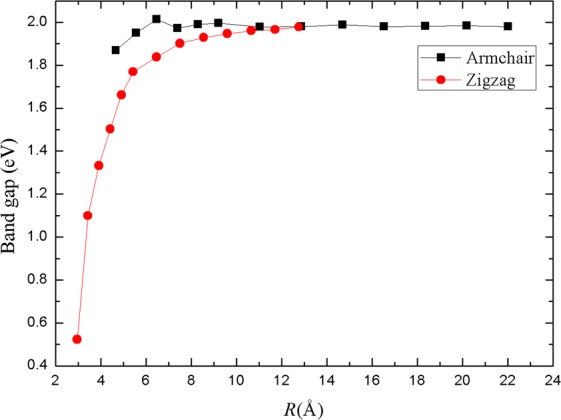
Band gap versus radius.

The dependence of band gap on radius for blue PNT is shown in Fig. 7. Interestingly, the band gap of blue PNTs are larger than black PNTs with the same radius^24^. When the radius is small, the band gap of zigzag blue PNTs rise sharply with the increase of radius. It can be seen that the band gap is rapidly increasing from 0.52 to 1.84 eV within 2.96 < *R* < 6.5 Å and then almost no more changes with the increase of radius. However, the band gap of armchair blue PNTs remain almost unchanged as the radius changes. In both cases, with the increasing radius, the band gap of nanotubes will be close to that of blue P sheet (2.07 eV). The variation trend of band gap with tube diameter is consistent with the results reported by Xiao *et al*.^34^.

**Figure 8:**
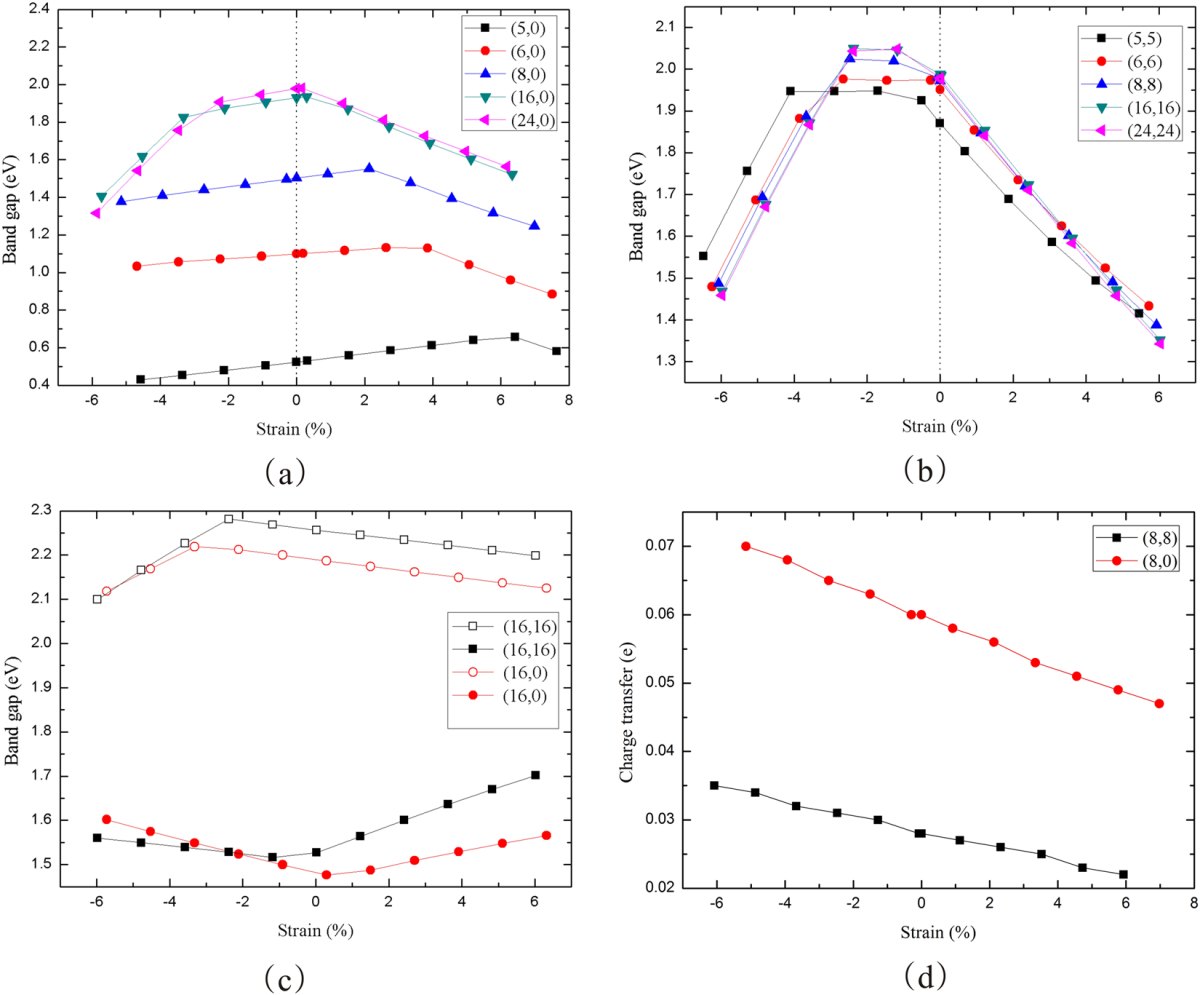
(**a**) Band gap of zigzag blue PNTs versus strain. (**b**) Band gap of armchair blue PNTs versus strain. (**c**) Variations of energy eigenvalues of the CBM and VBM as functions of the strain. (**d**) Charge transfer of two types of blue PNTs versus strain.

The axial strain (δ) has a significant effect on the electronic properties of blue PNTs, similar to the black phosphorus^41^. As shown in Fig. 8a, the band gap of zigzag blue PNTs (n = 5, 6, 8) exhibit a linear response to the axial strain between −2% and 2% when the radius is small. The maximal band gap increases with the increasing of n. For n = 5, 6, 8 nanotubes, the maximum values are at δ equal to 6.4%, 2.6% and 2.1%, respectively. As the radius increases (n > 16), the maximal band gap of blue PNTs is obtained in the pristine cases with *ε* = 0 and the curves almost coincide. However, for armchair blue PNTs, the band gap nonlinearity decreases when the axial strain changes from −2% to 2%, and the maximum band gap exists when the compressive strain is near −2% (see Fig. 8b). In addition to n = 5, the band gap increases and decreases linearly with the increase of strain in the −6.2 < δ < −2.5 and 0 < δ < 6.1, and the band gap values under each strain are almost identical. These properties are different with the phosphorene nanoribbon which exhibits a linear response to the tensile strain range from −4% to 4%^42^. The results for nanotubes (16, 0) and (8, 8) are in agreement with those for APNT-12 and ZPNT-8 in ref.^34^, which also proves the reliability of our calculation results. The valence band maximum (VBM) and conduction band minimum (CBM) with strain between −6% and 6% are plotted in Fig. 8c. It can be seen that VBM shift is the main reason for the band gap modification when the strain changes from −2% to 2%. The VBM shift cause the band gap difference between the two types of PNTs. This is mainly due to the P-P bonding states when the axial strain compresses or expands. Figure 8d shows that the charge transfer from the outer shell to the inner shell decreases linearly as the strain increases from −6% to 6%. This is owed to the covalent interactions as the P-atoms approach when the axial strain decreases.

**Figure 9:**
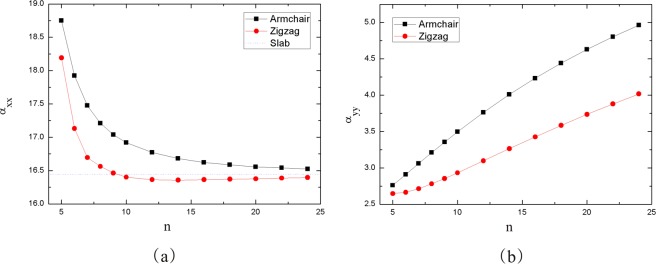
(**a**) α_xx_ and (**b**) α_yy_ per P as a function of n for two types of nanotubes.

The longitudinal (α_xx_) and transverse (α_yy_ = α_zz_) polarizabilities of nanotubes as a function of the radius were investigated with the Coupled Perturbed Hartree-Fork (CPHF) method. x and y are periodic directions in monolayer plane. For nanotubes, the periodic direction is x axis, y and z are the directions perpendicular to the tube axis (x). The results are shown in Fig. 9. Within 5 ≤ n ≤ 24, α_xx_ of both nanotubes decrease rapidly and approach a limiting value. α_yy_ increases slowly and converges gradually with the increase of n. Using polynomial fitting $\alpha {\mathtt{=}}{{\rm{c}}}_{{\mathtt{0}}}{\mathtt{+}}{{\rm{c}}}_{{\mathtt{1}}}{\mathtt{/}}{\mathtt{n}}{\mathtt{+}}{{\rm{c}}}_{{\mathtt{2}}}{\mathtt{/}}{{\mathtt{n}}}^{{\mathtt{2}}}{\mathtt{+}}{{\rm{c}}}_{{\mathtt{3}}}{\mathtt{/}}{{\mathtt{n}}}^{{\mathtt{3}}}$, we obtain α_xx_ = 16.39 and 16.25 when n tends to infinity for armchair and zigzag nanotubes, respectively. They are consistent with the values of the monolayer structure α_xx_ = 16.44. Similarly, we can get the limit value of α_yy_ by polynomial fitting. The results of the armchair and zigzag nanotubes are 8.02 and 7.05, respectively.

## Conclusions

In conclusion, we have investigated the mechanical, electronic and dielectric properties of blue PNTs by using first-principles calculations based on the DFT. The structure of armchair and zigzag blue PNTs with different radius are optimized. The formation energy, structural parameters, charge transfer, Young’s modulus, Poisson’s ratio, band gap and static electronic polarizabilities of the two types of PNTs have been computed. We obtain that the relationship between formation energy and radius follows *R*^−2^ law. Our results show that blue P nanotubes are expected to be stiffer than the armchair direction for black P nanotubes, having a Young’s modulus of approximately 136 GPa when the radius is large enough. The Poisson’s ratio of blue phosphorus nanotubes is smaller than that of black phosphorus nanotubes, but the value is closer to that of graphene. These prove that the properties of a single blue P sheet in these two directions (armchair and zigzag) are isotropic, in this respect they are similar to graphene. In addition, we study the static electronic polarizabilities with tube radius. For larger radius tubes, the longitudinal polarizability is more than twice the transverse components. This study provides a physical insight into the radius dependence of the mechanical and dielectric properties of blue P nanotubes.

### Computational methods

All calculations were carried out by using 3D-periodic density functional theory with Gaussian basis sets as implemented in the CRYSTAL simulation package^43^. The generalized gradient approximation (GGA) in the form of Perdew-Burke-Ernzerhof (PBE) is adopted for the exchange-correlation potential^44,45^. An effective core pseudo-potential(ECP) type basis set has been used^46^. The exponents of the most diffuse *sp* and *d* shells have been reoptimized in the present work. In the Brillouin zone, a Pack-Monkhorst *k* net with 8 × 8 × 8 points was used. The level of numerical accuracy was increased over the default settings of the software as described by Noel *et al*.^47^, selecting tolerances for bielectronic coulomb and Hartree-Fork exchange sums with keyword TOLINTEG 7 × 7 × 7 × 9 × 30. The SCF convergence threshold on zeroth-order energy is set to 10^−8^ Hartree. The keyword FMIXING = 80% is used to facilitate the convergence of the SCF iteration.
